# A Randomized Clinical Study Comparing Patient Satisfaction and Clinical Outcomes Associated With Two Nasal Packs Used in Nasal Surgery

**DOI:** 10.7759/cureus.108816

**Published:** 2026-05-13

**Authors:** Muhammad Junaid Farrukh, Kushagra Khanna, Mogana Rajagopal, Abdul Azim Al-Abrar Ahmad Kailani, Ilmi Akmaludin Mohamad, Raziin Zainal, Por Choo Shiuan, Tavanesan Mahindramany

**Affiliations:** 1 Faculty of Pharmaceutical Sciences, UCSI University, Kuala Lumpur, MYS; 2 Department of Otorhinolaryngology-Head and Neck Surgery, Faculty of Medicine, Universiti Teknologi MARA, Sungai Buloh, MYS; 3 Department of Otorhinolaryngology-Head and Neck Surgery, Hospital Al-Sultan Abdullah, Universiti Teknologi MARA, Puncak Alam, MYS

**Keywords:** biodegradable packing, haemostasis, mucosal healing, patient satisfaction, septoplasty

## Abstract

Objective

This study aimed to compare the efficacy, safety, and tolerability of two biodegradable nasal packs VelNez® (Datt Mediproducts Pvt. Ltd., New Delhi, India) and NasoPore® (Polyganics B.V., Groningen, the Netherlands) in patients undergoing nasal surgery, with the primary objective of assessing haemorrhage control time and biodegradability.

Methods

In this prospective, open-label, single-centre, randomized clinical trial, 20 patients undergoing nasal surgery were randomly assigned to receive either VelNez® (n=10) or NasoPore® (n=10). Primary endpoints were haemorrhage control time and nasal pack fragmentation at seven days post-surgery. Secondary endpoints included patient comfort, pain scores, endoscopic findings, and adverse events. Statistical analyses were conducted using t-tests and chi-squared tests with significance set at p<0.05.

Results

VelNez® showed significantly faster haemorrhage control (mean 2.6 minutes) compared to NasoPore® (mean 3.8 minutes; p=0.033). Fragmentation analysis revealed VelNez® had significantly less residue after seven days (p<0.01), indicating better biodegradability. Patients treated with VelNez® reported lower pain scores during surgery, discharge, and first follow-up (p<0.05) and experienced less nasal obstruction and discharge. Endoscopic examination showed less postoperative edema in the VelNez® group at seven days (p=0.019). No significant infections, allergic reactions, and fibrosis were observed in either group.

Conclusion

The VelNez® nasal pack demonstrated improved haemorrhage control, biodegradability, and patient comfort compared to NasoPore® in nasal surgery. Its use may improve postoperative outcomes, reduce patient discomfort, and lower health risks associated with nasal packing, contributing positively to public health by enhancing recovery and reducing complications.

## Introduction

Septoplasty may be performed either as a standalone procedure or in combination with other interventions such as functional endoscopic sinus surgery (FESS), rhinoplasty, or turbinoplasty. Among the array of strategies aimed at averting postoperative issues, the incorporation of nasal packing has emerged as a contentious point among surgeons [1]. Nasal packing plays an important role in preventing postoperative complications such as bleeding, septal haematoma, and synechiae formation while also facilitating the approximation of the mucoperichondrial flap and stabilization of the septal cartilage [2]. In addition, nasal packs promote mucosal healing, reduce the risk of mucosal adhesions, and help restore normal mucociliary clearance following sinus surgery. An ideal nasal pack should allow easy insertion and removal while minimizing pain and discomfort [3]. The primary benefit of postoperative nasal packing lies in its tamponade effect, which helps prevent surgical complications [4]. However, nasal packing is associated with several inherent disadvantages, including pain, bleeding, nasal mucosal damage, septal perforation, allergic reactions, sleep-related respiratory disturbances, and reduced arterial oxygen saturation during sleep [5]. In addition, patients often perceive pack removal as the most unpleasant aspect of the procedure [1], and nasal packing has been described as one of the most objectionable components of postoperative care [6]. Prolonged packing may further increase the risk of toxic shock syndrome. Moreover, in patients with obstructive sleep apnea, nasal obstruction due to packing can result in critically low oxygen saturation levels, necessitating close monitoring [7]. To address these limitations, various materials have been developed, including removable and absorbable nasal packs, leading to a significant expansion in available options. Biodegradable nasal packs have emerged as a promising alternative, as they do not require removal and begin to dissolve within a few days, allowing clearance through irrigation or suction [8].

VelNez® nasal pack (Datt Mediproducts Pvt. Ltd., New Delhi, India) is a biodegradable composite that fragments within a few days, eliminating removal and reducing patient discomfort. It undergoes in situ degradation, promoting haemostasis and reducing fibrosis. Previous studies report rapid haemostasis within minutes, good pain control, and minimal complications, supporting its safety and efficacy as a nasal dressing [9,10]. It has also been shown to degrade within 4-5 days with good tolerability and no significant adverse events, with similar safety observed in ear surgery [11]. Comparative studies indicate biodegradable packs like VelNez® and Rapid Rhino® (ArthroCare Corporation, Austin, TX, USA) provide faster haemostasis and better comfort than Merocel® (Medtronic, Minneapolis, MN, USA) [12].

NasoPore® (Polyganics B.V., Groningen, the Netherlands) is a biodegradable polyurethane foam nasal packing material composed of a hydrophilic absorptive component and a supportive scaffold. It conforms to the nasal cavity to provide effective haemostasis and gradually degrades in situ through hydrolysis, eliminating the need for removal and reducing patient discomfort. Previous studies have shown that NasoPore® improves postoperative comfort compared to non-absorbable nasal packing, with reduced pain, lower pressure sensation, and better early mucosal healing while maintaining comparable complication rates. Its use in dacryocystorhinostomy also reduces early re-bleeding and discomfort without affecting long-term anatomical or functional outcomes [8,13,14].

The present study aimed to compare VelNez® nasal packs with NasoPore® in terms of efficacy, feasibility, patient comfort, and need for repacking post-removal in patients undergoing nasal surgeries. The primary outcome variable was bleeding time and fragmentation time, and the secondary outcome variable was patient comfort.

## Materials and methods

Design

This was a prospective, open-label, single-centre, two-arm, randomized clinical study to compare patient satisfaction and clinical outcome associated with two nasal packs in nasal surgery.

Participants

For the study, 20 subjects were enrolled. All potential subjects of both genders and patients of all socioeconomic status who met the study-related inclusion and exclusion criteria were included (Table 1). The study was registered on ClinicalTrials.gov with the ID NCT07560254 and was conducted at the Department of Otorhinolaryngology-Head and Neck Surgery, Hospital Al-Sultan Abdullah, Universiti Teknologi MARA, Puncak Alam, Selangor, Malaysia. The clinical phase spanned approximately seven months, from the first patient first visit (FPFV) on September 11, 2024, to the last patient last visit (LPLV) on April 11, 2025.

**Table 1:** Inclusion criteria and exclusion criteria of the study LAR: legally authorized representative; HIV: human immunodeficiency virus; HCV: hepatitis C virus; VDRL: venereal disease research laboratory; HBsAg: hepatitis B surface antigen

Inclusion criteria
Subject eligible for the use of a nasal pack (either VelNez® or NasoPore®) in routine clinical practice after a planned nasal surgery; males and females aged 18 years and above; subjects or their LAR who can provide an informed consent form in writing; subjects who allow their study data to be collected at a predefined follow-up period
Exclusion criteria
Subjects who were not suitable for treatment with either VelNez® or NasoPore® nasal pack in routine clinical practice after a planned surgery; subjects/legal guardian (LAR) who cannot provide informed consent in writing; subject unwilling or unable to comply with the postoperative visits necessary for data collection; subject with an active infection at the surgery site; subject with a history of asthma; pregnant or lactating females; subjects who are on aspirin or anti-platelet drug therapy; subject positive for HIV, HCV, VDRL, and HBsAg; subjects who are allergic (hypersensitive) to any of the ingredients of the nasal packs; subject with bleeding disorders; any medical condition that, in the opinion of the investigator, would make the subject unsuitable for inclusion (e.g., a chronic, relapsing, or hereditary disease that may interfere with the outcome of the study); tumor cases

Sample size

This study was designed as a pilot/feasibility post-market clinical investigation with an exploratory primary objective. A formal a priori power calculation was not mandated. The sample size was determined based on published literature and feasibility considerations to generate preliminary comparative data in the Malaysian population. Using published data [9], which reported an expected proportion of postoperative pain complaints of approximately 34.24% (p=0.3424) with biodegradable nasal packing materials, the sample size was calculated using the formula $\mathrm{n}=(\mathrm{Z}^{2}\times\mathrm{p}\times(1-\mathrm{p}))/\mathrm{d}^{2}$.

Assuming a 95% confidence level (Z=1.96) and a 15% margin of error (d), the calculated minimum sample size was approximately 20 subjects. Accordingly, 20 subjects were enrolled and randomized in a 1:1 ratio, with 10 subjects allocated to each treatment arm (VelNez® and NasoPore®). The sample size of 20 subjects (10 per arm) is consistent with published pilot studies of nasal packing devices and is sufficient to detect clinically meaningful differences in the primary endpoint (haemorrhage control time) at an alpha level of 0.05, assuming a Cohen's d effect size of ≥0.8 (large effect), with approximately 60-70% power. The study was explicitly designed as a pilot study to provide preliminary Malaysian data to inform larger confirmatory trials.

Participant recruitment and randomization

**Figure 1:**
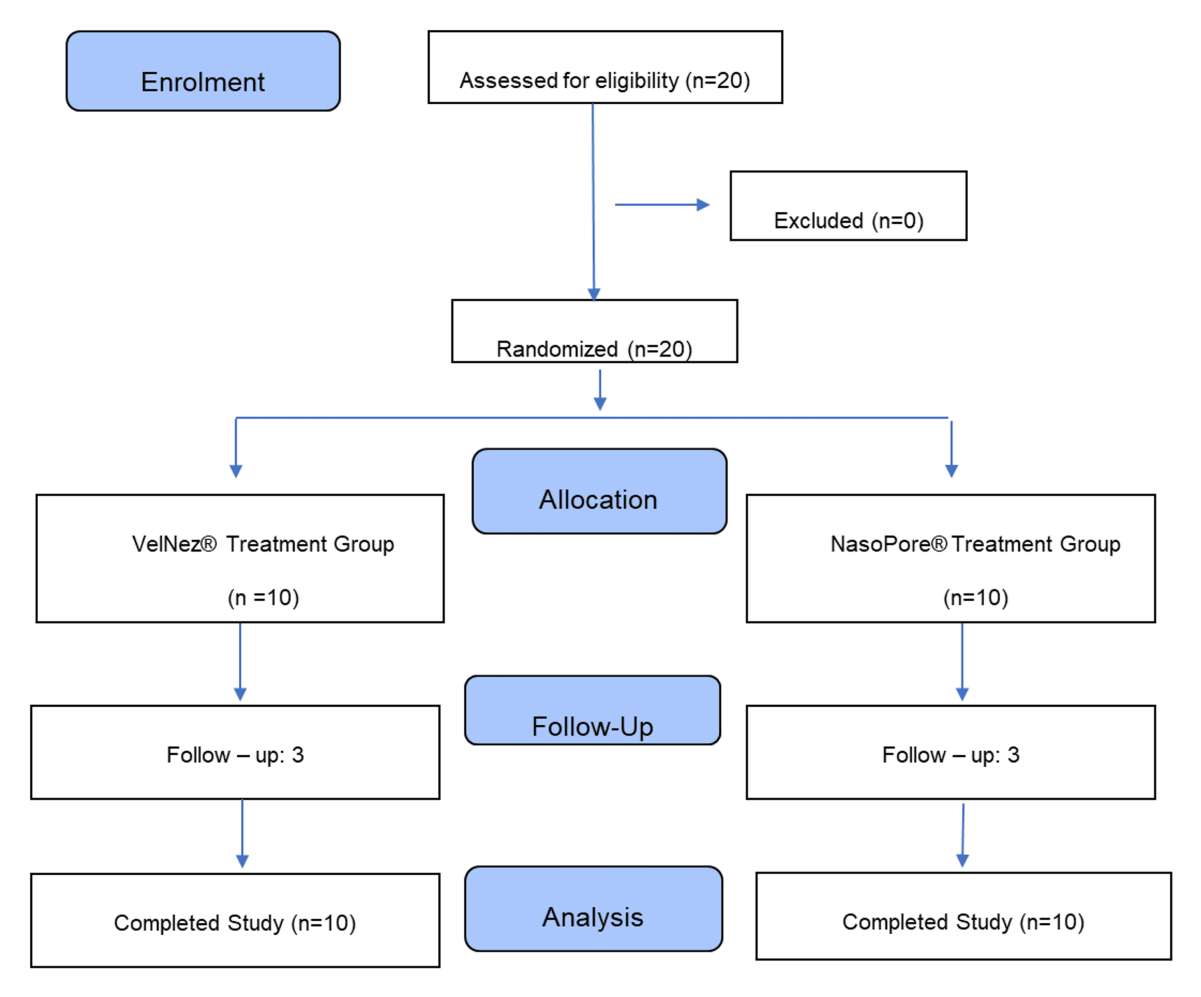
CONSORT diagram of clinical study CONSORT: Consolidated Standards of Reporting Trials

A list of patients planned to undergo nasal surgery was obtained. The patients were randomly assigned to receive VelNez® or NasoPore®. Randomization in this two-arm study was achieved using R Version 4.3.0 (R Foundation for Statistical Computing, Vienna, Austria). Each subject was assigned an equal probability of being placed in either treatment arm, thereby maintaining balance and minimizing selection bias. The process involved numbering the subjects sequentially from 1 to 20, followed by the use of a random number generator to assign each subject to one of the two treatment groups. The resulting allocation schedule assigned 10 subjects to the VelNez® group and 10 subjects to the NasoPore® group, ensuring an equal distribution across both arms. The patients and the observer were not informed of which nasal packing they had received. A unique ID was allotted to the study participants to maintain identity confidentiality (Figure 1).

Surgery and follow-up

Subjects scheduled for planned nasal surgery requiring nasal packing, such as septoplasty with or without FESS or turbinoplasty, were enrolled after screening and obtaining written informed consent at visit 1. Each participant was assigned a unique identification number to maintain confidentiality. Visit 2 involved the surgical procedure and application of the nasal packing device according to the randomization schedule. This was followed by three mandatory follow-up visits, namely, the first at seven days (F1), the second at 14 days (F2), and the final at 90 days (F3) post-surgery, during which safety, healing, and device-related outcomes were assessed. Telephonic follow-ups were conducted for participants who missed any scheduled visits to ensure complete data collection.

Intervention

VelNez® nasal pack, manufactured by Datt Mediproducts Pvt. Ltd. and marketed by AirMed DWC LLC, Malaysia, is a biodegradable composite intended for nasal packing that fragments within a few days after application. As it does not require manual removal, it may help avoid the discomfort typically associated with the removal of conventional nasal packs. It is designed to support haemostasis and may aid in mucosal healing while minimizing fibrosis.

NasoPore® is a biodegradable synthetic polyurethane foam used in the present study. Its polyurethane structure provides initial mechanical support through compressive strength, while the hydrophilic component absorbs blood or fluids and gradually fragments over time.

Study endpoints

Primary Efficacy Endpoint

The primary efficacy endpoint was defined as a composite measure integrating both safety and clinical effectiveness outcomes. It included the proportion of study participants who achieved complete degradation of the nasal pack within seven days without any associated adverse events. Additionally, fibrosis levels at the surgical site were assessed during follow-up visits. Scar formation was evaluated alongside the incidence and severity of pain, with particular attention to moderate to severe pain among participants. The endpoint also incorporated the incidence of high-pressure effects resulting from nasal pack application, specifically focusing on patient-reported breathing discomfort.

Secondary Efficacy Endpoints

The secondary efficacy endpoints included haemorrhage control time, defined as the duration (in minutes) from application of the nasal pack at the completion of surgery to the achievement of complete haemostasis, as assessed by the operating surgeon. Further evaluations included adhesion status and endoscopic findings, such as edema, nasal secretions, and the presence of polyps, across follow-up visits. Additional patient-centred outcomes comprised pain assessment using the visual analogue scale (VAS) at predefined time points and subject comfort parameters including pressure sensation, nasal obstruction, nasal discharge, and sleep disturbance. The residue and fragmentation profile of the nasal pack were also observed during follow-up, alongside surgeon-reported questionnaire ratings and overall subject satisfaction scores.

Ethical consideration

This study received ethical approval from the Universiti Teknologi MARA (UiTM) Research Ethics Committee (approval number: REC/09/2024 (ST/CT/9)), ensuring that the study adhered to established ethical standards and guidelines. Prior to enrolment, all patients were provided with comprehensive information about the study's purpose, procedures, potential risks, and benefits in a clear and understandable manner. Adequate time was given for patients to consider their participation, and written informed consent was obtained from each participant before any study-related activities commenced. Confidentiality of patient information was maintained throughout the study, and participants were assured of their right to withdraw at any time without affecting their medical care.

Data analysis

The collected data were analyzed using both descriptive and inferential statistical methods. Descriptive statistics summarized demographic characteristics using percentages, means, and standard deviations (SD). Parametric tests such as the t-test and analysis of variance (ANOVA) were applied to numerical variables including pain score, subject comfort scale, bleeding time, haemorrhagic control, and fragmentation time to determine mean differences and correlations. For categorical variables such as bleeding status, allergic reactions, adhesion status, severity of infection, and adverse events, the chi-squared test was used to evaluate associations. Statistical significance was set at p<0.05.

## Results

Demographic data

A total of 20 patients were enrolled in the study. The mean (SD) age of the participants was 37.20 (10.36) years. Regarding gender, 12 patients (60%) were male, and eight patients (40%) were female. The mean height of the patients was 162.0 cm, with an SD of 5.94 cm, reflecting some variability in height. The average weight was 75.08 kg, with an SD of 11.7 kg, indicating a broad range of body weights among the participants. In terms of smoking status, only one patient (5%) was a smoker, while 19 patients (95%) were non-smokers. Patients' sociodemographic characteristics between the groups are shown in Table 2.

**Table 2:** Sociodemographic characteristic difference between the two groups Values are presented as mean (SD) for continuous variables and number (percentage) for categorical variables. Percentages are calculated within each group. FESS: functional endoscopic sinus surgery

Variables	VelNez® (n=10)	NasoPore® (n=10)
Age, mean (SD), years	38.50 (12.28)	35.90 (8.47)
Gender, n (%)		
Male	5 (50)	7 (70)
Female	5 (50)	3 (30)
Height, mean (SD), cm	162.0 (5.94)	163.7 (6.35)
Weight, mean (SD), kg	75.08 (11.7)	74.97 (22.2)
Smoking status, n (%)		
Yes	0 (0)	1 (10)
No	10 (100)	9 (90)
Type of surgery, n (%)		
Septoplasty with FESS	0 (0)	1 (10)
Turbinoplasty	2 (20)	3 (30)
Septoplasty with FESS+turbinoplasty	5 (50)	4 (40)
Septoplasty without FESS	3 (30)	2 (20)

Haemorrhage control time

**Figure 2:**
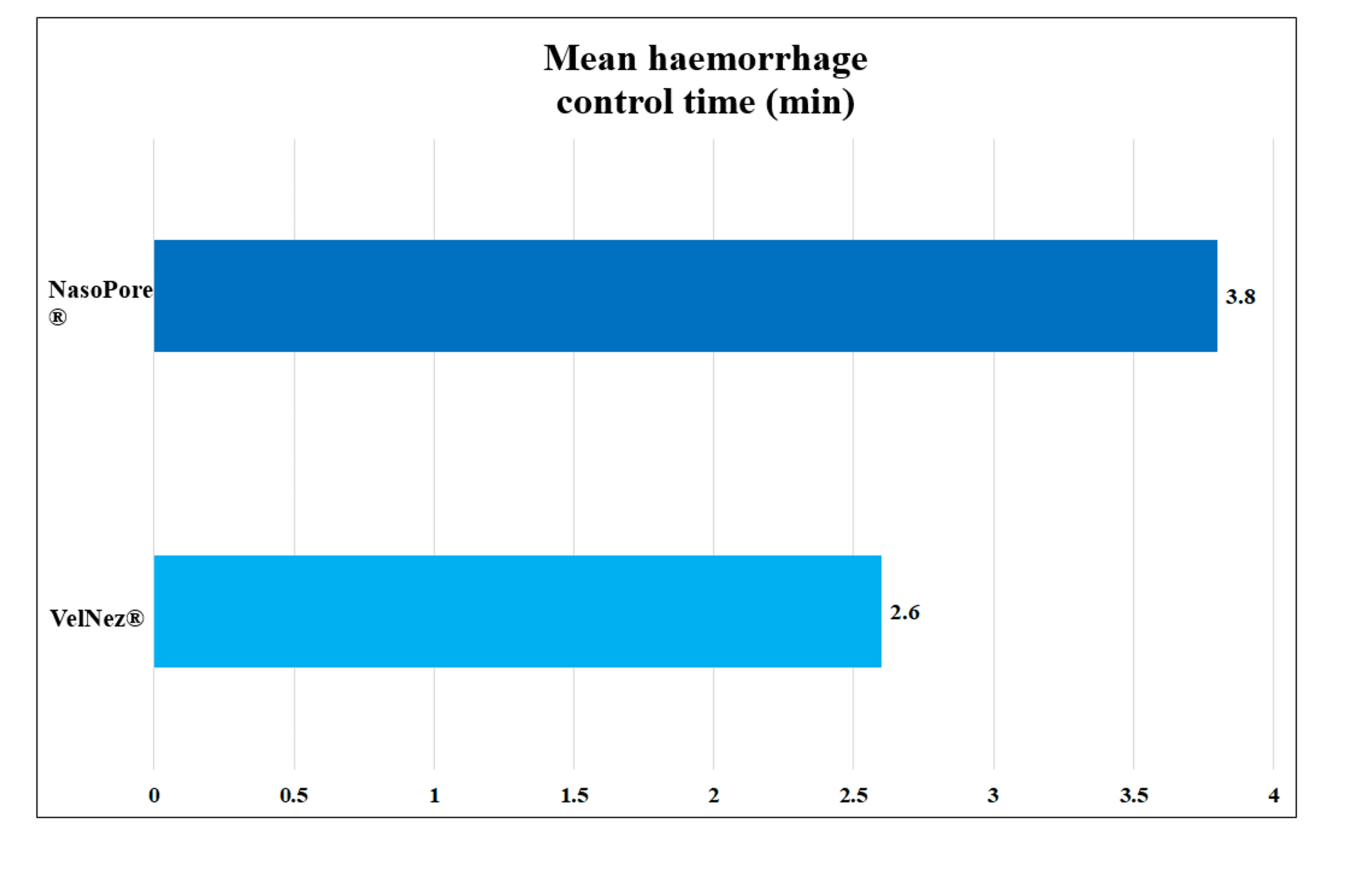
Comparison of mean haemorrhage control time between VelNez® and NasoPore® Data are presented as mean±SD. Statistical significance was considered at p<0.05.

VelNez® achieved a significantly shorter haemorrhage control time compared to NasoPore®, with mean times of 2.60 and 3.8 minutes, respectively (p=0.033). Figure 2 presents a comparison of the mean haemorrhage control times between the two nasal pack treatments, VelNez® and NasoPore®.

Fragmentation time/residue remaining after seven days

**Figure 3:**
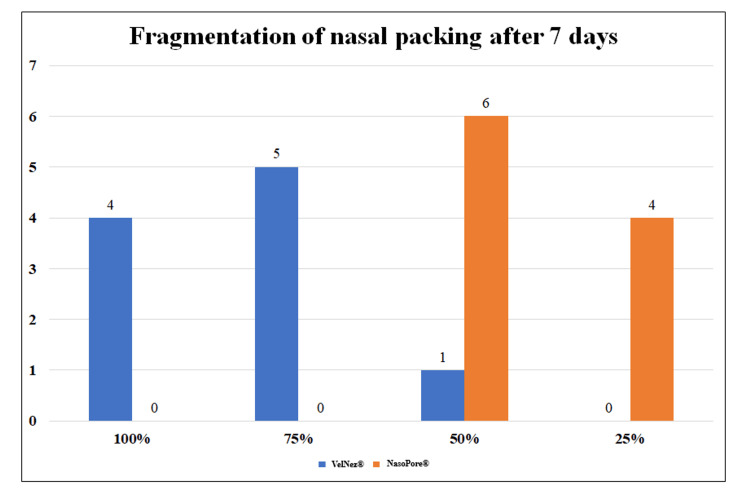
Comparison of fragmentation of nasal packing after seven days between VelNez® and NasoPore® Data are presented as n (%). Statistical significance was considered at p<0.05.

VelNez® is more likely to break down fully, leaving minimal or no residue, whereas NasoPore® retains a significantly higher proportion of residue after seven days (p<0.01). After seven days, VelNez® nasal packing showed quicker fragmentation, with four cases exhibiting complete (100%) fragmentation and five cases at 75% fragmentation. Only one case showed 50% fragmentation. In contrast, NasoPore® packing demonstrated slower fragmentation, with no cases of complete or 75% fragmentation, but six cases at 50% and four cases at 25% fragmentation. Comparison of fragmentation time between Velez and NasoPore® is shown in Figure 3.

Surgeon questionnaire

NasoPore® demonstrated superior performance in usability-related parameters, achieving the unanimous highest ratings for ease of handling (100%) and ease of application (100%), compared to VelNez® (90% and 80%, respectively). VelNez® showed a marginally higher rating in conformance to tissue surfaces, with 90% of responses in the top category versus 80% for NasoPore®. Regarding overall device performance, NasoPore® was rated more favorably (90%) than VelNez® (80%). Collectively, both devices were well accepted; however, NasoPore® appears to provide enhanced usability, whereas VelNez® may offer a slight advantage in tissue conformity (Table 3).

**Table 3:** Comparison of surgeon questionnaire between VelNez® and NasoPore® Score 1: best/most favorable; Score 2: less favorable

Parameter	VelNez®		NasoPore®	
	1	2	1	2
Ease of handling	9 (90%)	1 (10%)	10 (100%)	0 (0%)
Appropriateness of instructions	2 (20%)	8 (80%)	3 (30%)	7 (70%)
Ease of application	8 (80%)	2 (20%)	10 (100%)	0 (0%)
Conformance to tissue surfaces	9 (90%)	1 (10%)	8 (80%)	2 (20%)
Overall performance	8 (80%)	2 (20%)	9 (90%)	1 (10%)

Endoscopy findings (polyp, edema, and secretions)

**Figure 4:**
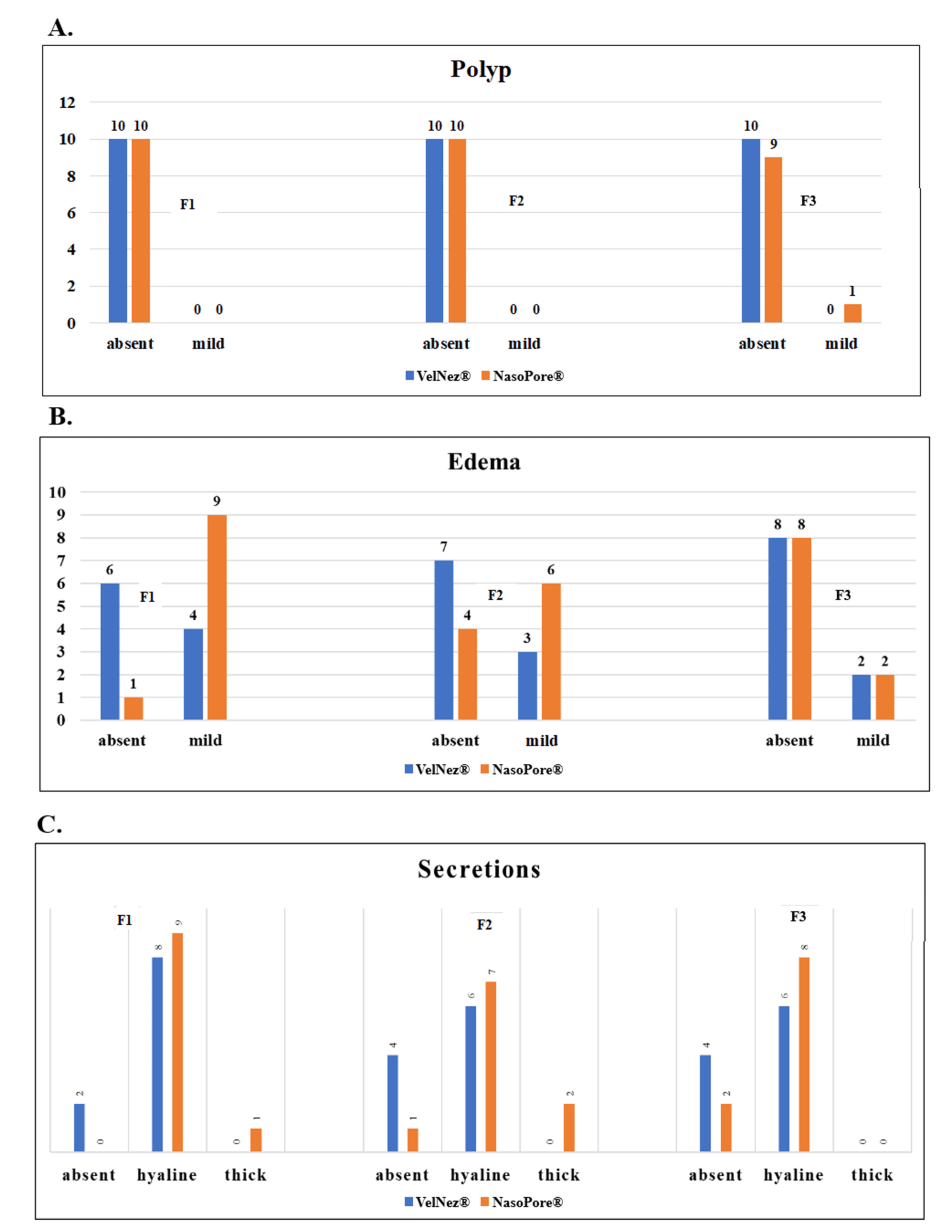
Endoscopic findings (a) Polyps, (b) edema, and (c) secretions in the VelNez® and NasoPore® groups at follow-up points F1-F3. Data are presented as the number of patients, n (%), in each category (absent, mild, hyaline, thick) at each time point. Statistical significance was considered at p<0.05.

Both brands indicate no polyps in F1 and F2, but NasoPore® detected mild polyps in one case during F3. During the first follow-up session (F1), VelNez® demonstrated significantly less mild edema compared to NasoPore® (p=0.019). Regarding secretions, VelNez® consistently exhibited a higher proportion of absent secretions across all time points, suggesting a comparatively cleaner profile. The details of the endoscopic findings are shown in Figure 4.

Assessment of tolerability

**Figure 5:**
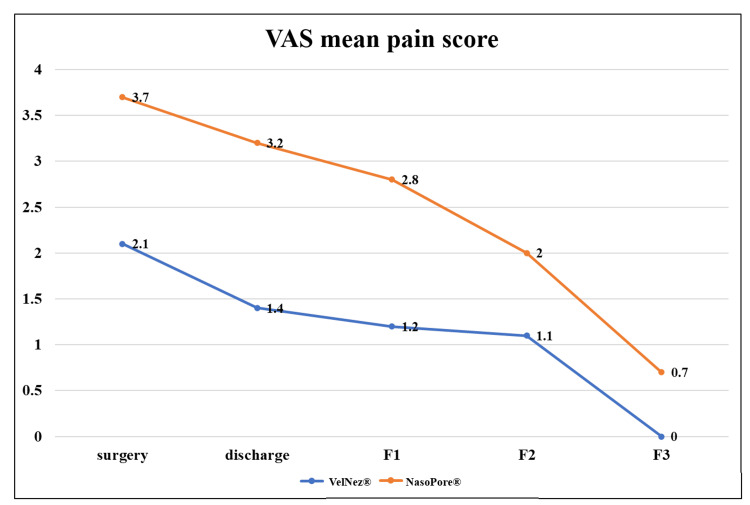
VAS mean pain scores between the two groups Mean VAS pain scores for VelNez® and NasoPore® groups at surgery, discharge, and follow-up points (F1-F3). VAS: visual analogue scale

The VAS pain scores for VelNez® were consistently lower than NasoPore® across all stages, with significant differences observed at surgery, discharge, and follow-up (F1). VelNez® provided more effective pain relief, as shown by its lower scores at these stages. By F3, both treatments resulted in minimal pain, but the difference was no longer significant. Overall, VelNez® demonstrated superior pain management compared to NasoPore®. The mean pain score trends between the two groups are shown in Figure 5.

Assessment of subject comfort

VelNez® showed better outcomes in several parameters. For pressure effect due to packing, VelNez® scores lower mean values at F2 (0.50 vs. 1.40), indicating less discomfort. Nasal discharge control and nasal obstruction scores were consistently lower in the VelNez® group at all follow-ups, particularly notable in nasal obstruction at F2 (0.90 vs. 1.40) and F3 (0.00 vs. 0.30), reflecting better symptom control. Sleep disturbance scores favor VelNez® at all points, especially at F2 (1.0 vs. 0.0), though the difference is small. Post-nasal drip shows lower mean values for VelNez® at F3 (0.10 vs. 0.40), indicating better control. Table 4 presents the assessment of subject comfort comparing two groups, VelNez® and NasoPore®, across multiple parameters at three different follow-ups (F1, F2, F3).

**Table 4:** Assessment of subject comfort between VelNez® and NasoPore® groups across follow-up periods Data are presented as mean±standard deviation (SD). Comparisons were made between the VelNez® and NasoPore® groups at follow-up time points F1, F2, and F3. The p-values indicate intergroup comparisons at each time point. Statistical significance was considered at p<0.05. "-" indicates not applicable or not calculated.

Parameter	Group	F1: mean (SD)	P-value	F2: mean (SD)	P-value	F3: mean (SD)	P-value
Pressure effect due to packing	VelNez®	2.60 (3.33)	0.26	0.50 (0.97)	-	0.00 (0.00)	0.155
	NasoPore®	3.50 (3.06)	-	1.40 (2.54)	-	0.00 (0.00)	-
Nasal discharge	VelNez®	3.60 (3.37)	0.250	1.10 (1.85)	0.102	0.30 (0.43)	0.190
	NasoPore®	4.60 (3.13)	-	2.40 (2.50)	-	1.20 (3.11)	-
Nasal obstruction	VelNez®	3.70 (3.43)	0.128	0.90 (1.37)	0.30	0.00 (0.00)	0.165
	NasoPore®	5.40 (3.02)	-	1.40 (2.6)	-	0.30 (0.94)	-
Difficulty in swallowing	VelNez®	2.20 (3.45)	0.47	0.60 (1.34)	-	0 (0)	0.08
	NasoPore®	2.10 (2.92)	-	0 (0)	-	0 (0)	-
Sleep disturbance	VelNez®	1.10 (2.80)	0.21	1.0 (2.53)	0.11	0 (0)	NA
	NasoPore®	4.30 (3.65)	-	0.0 (0.0)	-	0 (0)	-
Post-nasal drip	VelNez®	1.20 (1.81)	0.60	1.30 (2.75)	0.226	0.10 (0.31)	0.116
	NasoPore®	3.10 (3.21)	-	0.60 (0.84)	-	0.40 (0.69)	-
Infection	VelNez®	0 (0)	0.165	0.80 (2.52)	0.165	0 (0)	0.165
	NasoPore®	0.80 (2.52)	-	0 (0)	-	0.60 (1.89)	-

## Discussion

Nasal surgeries typically require nasal packing to achieve haemostasis. A wide range of nasal packing materials has been developed over time, and the choice of material plays a crucial role in postoperative wound healing [15]. VelNez® and NasoPore® are commonly used nasal packing materials in current clinical practice. The present study compared these materials in terms of efficacy, feasibility, and patient comfort. VelNez® demonstrated a significantly shorter haemorrhage control time than NasoPore® and showed more complete fragmentation with minimal residue, whereas NasoPore® retained a higher proportion of residue after seven days (p<0.01). This suggests that VelNez® undergoes faster and more extensive degradation compared to the slower, partial fragmentation observed with NasoPore®. Absorbable nasal packing materials are essential for achieving haemostasis and promoting wound healing by providing a stable environment, preventing adhesions, and supporting tissue repair. Their key advantage lies in eliminating the need for removal, thereby reducing patient discomfort and associated complications [16,17]. These findings are consistent with previous studies reporting improved patient acceptability and reduced discomfort with biodegradable nasal packs, particularly due to the avoidance of painful removal [10]. While materials such as NasoPore® have demonstrated effective haemostasis [18], differences among biodegradable packs are often more evident in the early postoperative period and tend to diminish over time. The faster disintegration of VelNez® may facilitate earlier mucosal regeneration and reduce clinical workload by eliminating removal procedures. It was also associated with reduced edema in the early postoperative period, although this difference was not sustained at later follow-ups, indicating comparable long-term performance. Similarly, no significant differences were observed in nasal secretions between the groups over time. Assessment of tissue healing parameters indicated favorable postoperative outcomes in both treatment groups, with no evidence of clinically significant fibrosis or abnormal scar formation at the surgical site in any subject throughout the study period. However, VelNez® was devoid of secretions, indicating a cleaner postoperative profile, whereas NasoPore® demonstrated relatively higher hyaline and thick secretions, suggesting slower clearance. These findings are supported by studies indicating that faster-degrading biodegradable materials improve mucosal healing and reduce secretion accumulation [15]. Additionally, retained absorbable material during gradual fragmentation may impair mucociliary clearance and contribute to persistent nasal discharge in the early postoperative period [19]. VelNez® was also associated with improved pain control, which is a critical determinant of postoperative recovery and patient satisfaction. This may be attributed to reduced mucosal trauma, lower packing pressure, and faster fragmentation. Consistently lower symptom scores for nasal obstruction, discharge, and post-nasal drip further indicate better patient comfort with VelNez® [9]. Although differences in sleep disturbance were modest, improved symptom control and lower pressure effects suggest reduced mechanical irritation and better respiratory function during recovery. Overall, VelNez® nasal packing appears to provide a more efficient and patient-friendly alternative to NasoPore®, particularly in the early postoperative period, with comparable outcomes observed over time.

Despite these encouraging findings, the present study has certain limitations. The study was conducted at a single centre with a relatively small sample size, which may limit the generalizability and statistical power of the results. In addition, the short follow-up duration may not fully capture long-term postoperative outcomes and complications. Therefore, larger multicentre studies with extended follow-up periods are warranted to further validate the clinical benefits of VelNez® nasal packing.

## Conclusions

VelNez® nasal packing demonstrated faster haemostasis and more complete and rapid degradation compared to NasoPore®, which may facilitate improved wound healing. It was associated with lower early postoperative edema and reduced symptom scores, suggesting better patient comfort. VelNez® also showed favorable trends in pain reduction and nasal symptoms such as obstruction and discharge. These advantages position VelNez® as a more effective and patient-friendly choice for nasal surgery postoperative care.
